# APE1 may influence CD4+ naïve T cells on recurrence free survival in early stage NSCLC

**DOI:** 10.1186/s12885-021-07950-1

**Published:** 2021-03-06

**Authors:** Yanping Li, Xiaolong Zhao, He Xiao, Bo Yang, Jie Liu, Wen Rao, Xiaoyan Dai, Mengxia Li, Nan Dai, Yuxin Yang, Dong Wang

**Affiliations:** 1 School of Nursing, Chongqing Medical and Pharmaceutical College, No. 82, Daxuecheng Rd, Shapingba Dist, Chongqing, 401331 China; 2 Cancer Center, Daping Hospital, Army Medical University (Third Military Medical University), No. 10 Changjiang Zhi Rd., Yuzhong Dist, Chongqing, 400042 China

**Keywords:** Non-small cell lung cancer, APE1, Tumor infiltrating lymphocytes, Prognosis

## Abstract

**Background:**

It was demonstrated that multifunctional protein APE1 (Apurinic/apyrimidinic endonuclease 1) is closely related to tumor immune microenvironment in a number of investigations, Meanwhile, the abundance of tumor infiltrating lymphocytes (TILs) has been shown as a prognosis indicator in some researches. However, it remains unclear whether APE1 is involved in the process of TILs affecting the prognosis of patients. To this end, we investigated the associations between APE1 and TILs in non-small cell lung cancer (NSCLC) and explored whether APE1 would influence the associations of CD4^+^ T cells infiltration with the prognosis of patients.

**Methods:**

Genome-wide expression datasets were obtained from the Gene Expression Omnibus (GEO) public database under accession number GSE68465, GSE30219, GSE31210 and GSE50081. MCPcounter and CIBERSORT analysis was conducted to evaluate the abundance of TILs in 1006 NSCLC patients of GEO database. Spearman correlation tests were used to evaluate correlations between abundance of various TILs and APE1 expression. RFS (recurrence free survival) was estimated using the Kaplan–Meier method and the Cox proportional-hazards model. The expression level of APE1 and tumor-infiltrating CD4^+^ T cells was evaluated by immunohistochemistry (IHC).

**Results:**

The results showed that the abundance of CD4^+^ naïve T cells was negatively associated with the APE1 expression. CD4^+^ naïve T cells infiltration was a favorable prognostic factor for RFS, however, there was no effect of CD4^+^ T cells infiltration on RFS in patients with high APE1 expression. Subsequently, it was further confirmed that CD4^+^ T cells infiltration was negatively associated with the APE1 expression level in 108 NSCLC tissue samples; high CD4^+^ T cells infiltration was associated with longer RFS in low APE1 expression group but not in APE1 high expression group.

**Conclusion:**

These results suggested that APE1 may affect the relationship between CD4^+^ T cells infiltration and prognosis in NSCLC. This study provides new insights into predictors of outcome in patients with NSCLC, and suggests that combining immunotherapy and APE1-targeted therapy may be a promising treatment for NSCLC.

**Supplementary Information:**

The online version contains supplementary material available at 10.1186/s12885-021-07950-1.

## Background

APE1 (apurinic/apyrimidinic endonuclease 1) is a ubiquitous multifunctional gene with the functions of base excision and redox regulation and strongly associated with prognosis in cancer patients [1]. The expression of APE1 is up-regulated in many kinds of tumors including non-small cell lung cancer (NSCLC) [2]. A large number of studies indicated that APE1 is related to the first-line chemotherapeutic outcomes in advanced non-small cell lung cancer (NSCLC) [3, 4]. Furthermore, higher expression of APE1 in cytoplasm is related to shorter cancer specific survival (CSS) and relapse free survival (RFS) [5]. Recent studies have shown that the serum APE1 level is related to the therapeutic effect of NSCLC [6, 7]. However, the potential mechanism of APE1 affecting the prognosis of patients has not been fully elucidated.

Distinct tissue immune microenvironments determine the disease progression and affect clinical outcome in NSCLC [8, 9]. The relationship between tumor infiltrating lymphocytes (TILs) and the prognosis of NSCLC has been extensively studied, not surprisingly, most of these studies suggested that high lymphocyte infiltration indicates the favorable prognosis [10–12]. Due to these reasons, the explorations of immunotherapy for lung cancer have been growing vigorously. APE1, thanks to its versatile functions, has been reported recently to be involved in a variety of immune responses [13]. APE1 was found to be a regulatory protein to participate in macrophage inflammatory response through regulating TNF-α, IL-6 and IL-12 [14]. Another study showed that APE1 can regulate the production and secretion of IL-12 from antigen presenting cells and control helper T (Th) cells immune response [15]. In addition, it was found that APE1 also can be a “tuning molecule” to regulate the proliferation of B cells through activating CD40 pathway [16]. However, these studies were confined to experiments in vitro and did not include the evaluation of patients’ prognosis. Thus, in the present study, we explored the relationship between APE1 and the abundance of TILs in several sets of NSCLC clinical samples to better understand the APE1-related immune microenvironment.

## Methods

### Patients and specimens

To analyze the relationship between APE1 expression and tumor infiltrating lymphocytes of NSCLC, four data sets GSE68465, GSE30219, GSE31210 and GSE50081 were included in this study in which genome-wide gene expression was all profiled with Affymetrix HG-U133 chips (Affymetrix). Raw CEL files were downloaded from GEO and “threesetp” in R package “affyPLM” was used to perform background adjustment, normalization and summarization and gene expression values were represented as log2 scale. Batch effects were corrected by the main function “VirtalArrayComBat” after merged by the probe IDs [17]. The probe with the largest interquartile range was selected when multiple probes corresponded to one gene ENTREZID. A total of 12,402 genes from 1006 patients were included in the analysis. Furthermore, 20 samples collected from NSCLC patients who underwent surgical resection in 2019 at Daping Hospital were subjected to transcriptome sequencing to evaluate genome-wide gene expression profile. Patients who underwent radical resection and histologically diagnosed with NSCLC from 2010 to 2013 at Daping Hospital were included in our validation set. All patients had provided written informed consent during hospitalization. The study was not only approved by the Ethics Committees of Daping Hospital (2017 number 30), but also was conducted in accordance with the Declaration of Helsinki.

### Human transcriptome array

Transcriptome analysis was performed to identify the genes expression of 20 samples. RNA was extracted from deparaffinized and proteinase K–treated FFPE tissue (20–40 mg) using RNA prep Pure FFPE Kit (QIAGEN, Valencia, CA, USA) according to the manufacturer’s instructions. RNA purity was checked using the NanoPhotometer® spectrophotometer (IMPLEN, CA, USA). RNA concentration was measured using Qubit® RNA Assay Kit in Qubit® 2.0 Flurometer (Life Technologies, CA, USA). A total amount of 2 μg RNA per sample was used as input material for the RNA sample preparations. Sequencing libraries were generated using NEBNext® UltraTM RNA Library Prep Kit for Illumina® (NEB, USA) following manufacturer’s recommendations and index codes were added to attribute sequences to each sample. The clustering of the index-coded samples was performed on a cBot Cluster Generation System using TruSeq PE Cluster Kit v3-cBot-HS (Illumia) according to the manufacturer’s instructions. HTSeq was used to count the reads numbers mapped to each gene. And then FPKM of each gene was calculated based on the length of the gene and reads count mapped to this gene. A total number of 14,772 genes were finally remained in the expression matrix.

### Evaluation of the abundance of TILs and scRANseq signatures

For GEO database, MCPcounter [18] method and FARDEEP [19] method were used individually to evaluate the abundance of immune cells. The basic algorithm adopted by MCPcounter was the geometric mean of immune cell transcriptomic markers called MCPcounter score whereas the FARDEEP was principally based on the overall gene deconvolution algorithm [19]. The LM22 reference matrix (NIHMS670442-supplement-2) accompanied with the method CIBERSORT originally proposed by Newman was used in FARDEEP as the reference matrix [20]. For our sequencing data, EPIC [21] method was used to estimate the abundance of immune cells as well as one uncharacterized cell type. The normalized count reads was used as the gene expression of the bulk samples and pre-compiled reference gene expression profiles as reference matrix in EPIC. To further characterize the more refined immune subpopulations potentially affected by the expression of APE1, two well-characterized signatures were exploited [22]. The CD8-C4-GZMK/CD8-C6-LAYN signal ratio and TNFRSF9+ Treg signature were proposed to measure the ratio of “pre-exausted” to exhausted T cells and recently activated Treg cells in the originally published article [22]. There were 21, 318 and 52 genes that remained to be included for calculation of CD8-C4-GZMK/CD8-C6-LAYN signal ratio and TNFRSF9+ Treg signature in the combined GEO dataset. In our own NSCLC RNAseq data, 25, 369 and 55 genes were retrieved for CD8-C4-GZMK, CD8-C6-LAYN signal ratio and TNFRSF9+ Treg signature. The CD8-C4-GZMK/CD8-C6-LAYN signal ratio were calculated as averaging the expression of genes in CD8-C4-GZMK divided by the mean expression of genes involved in the CD8-C6-LAYN. The TNFRSF9+ Treg was estimated through the expression average of genes in TNFRSF9+ Treg signature. The data can be obtained in the supplementary files.

### Prognosis evaluation

For GEO data set, clinical data were acquired from the publicly available data. For our validated data set, clinical data were acquired from medical records and telephone follow-up. Recurrence-free survival (RFS) was used as time to event endpoint. RFS was defined as the time from surgery to first documentation of disease recurrence.

### Immunohistochemistry (IHC)

Immunohistochemistry and corresponding evaluation were performed as described previously [23]. Briefly, Parafn-embedded tissues were cut into about 3 μm sections and spread on glass slides. The sections were deparafnized and rehydrated through graded alcohols, and then antigen retrieval by high pressure heating streamer using Tris/EDTA buffer (heat induced epitope retrieval). After passing on to cool, the sections were treated with 3% H_2_O_2_-methanol solution for 10 min to eliminate endogenous peroxidase activity. The sections were then washed in PBS and incubated overnight at 4 °C with a primary antibody: APE1 (SC-17774, Santa Cruz Biotechnology Inc.), CD4 (ZA-0519, ZSGB-BIO). After washing with PBS, the sections were incubated with a horseradish peroxidase-conjugated secondary antibody (Rabbit HRP EnVision TM+, Dako, Denmark) for 30 min at 37 °C. After washing with PBS, the sections were incubated with 3, 3-diaminobenzidine (DAB) substrates for 3 min, counterstained with hematoxylin for 2 min, and finally dehydrated. The brown color indicated positive staining. For the negative control, the steps were the same except that the primary antibody was ruled out. IHC staining score of APE1 was determined based on the staining intensity of cancer cells and graded as follows: negative, score 0; weak, score 1; moderate, score 2; and strong, score 3. Score 2 and 3 were defined as high expression, whereas score 0 and 1 were defined as low expression. To evaluate the infiltration of CD4^+^ T cell, five microscope fields were randomly selected, and the number of positive stained cells and nucleated cells in each field was calculated by Image-Pro Plus software 6.0. Tumor-infiltrating CD4^+^ cells were classified as high (≧ 20%) and low (< 20%) infiltration (supplementary Table 1).

### Statistical analyses

All statistical analyses were performed using GraphPad Prism software 7.0 (GraphPad Software, Inc.) or statistical package SPSS (IBM SPSS Statistics for Windows, Version 19.0). Spearman correlation tests were used to evaluate correlations between abundance of various TILs and APE1 expression. Cox proportional hazard regression analysis was performed to evaluate the prognosis of the abundance of various TILs, APE1 expressions and other clinicopathological charactersitics for RFS. Multivariate Cox regression was used to determine independent prognostic factors for RFS. Comparisons of proportions of high CD4^+^ naïve T cells among subgroups with different APE1 expression were determined by Chi-square test. In Kaplan Meyer analysis, comparisons of RFS were performed by Log-rank test. Multivariate Cox regression was used to test interaction for RFS between subgroups categorized with APE1 expression and abundance of CD4^+^ naïve T cells as continuous variable. *P* < 0.05 was considered statistically significant.

## Results

### Patients’ characteristics

Combined dataset from four GEO database GSE68465, GSE30219, GSE31210 and GSE50081 comprised of a total of 1006 NSCLC cases. The clinical information in the whole population including age, sex and clinical stage was shown in Table 1.

**Table 1:** Baseline characteristic of four merged GEO datasets

Characteristic	The number of patients, n (%)
Age	63.15 ± 10.30
Age range	
≥ 60 years	675 (67.10)
< 60 years	331 (32.90)
Sex	
Male	480 (47.71)
Female	526 (52.29)
Histological cell type	
Adenocarcinoma	765 (76.04)
Squamous cell carcinoma	101 (10.04%)
Others	140 (13.92)
Tumor stage	
I + II	907 (90.16)
III + IV	98 (9.74)

### Correlation between APE1 expression and TILs infiltration

**Fig. 1:**
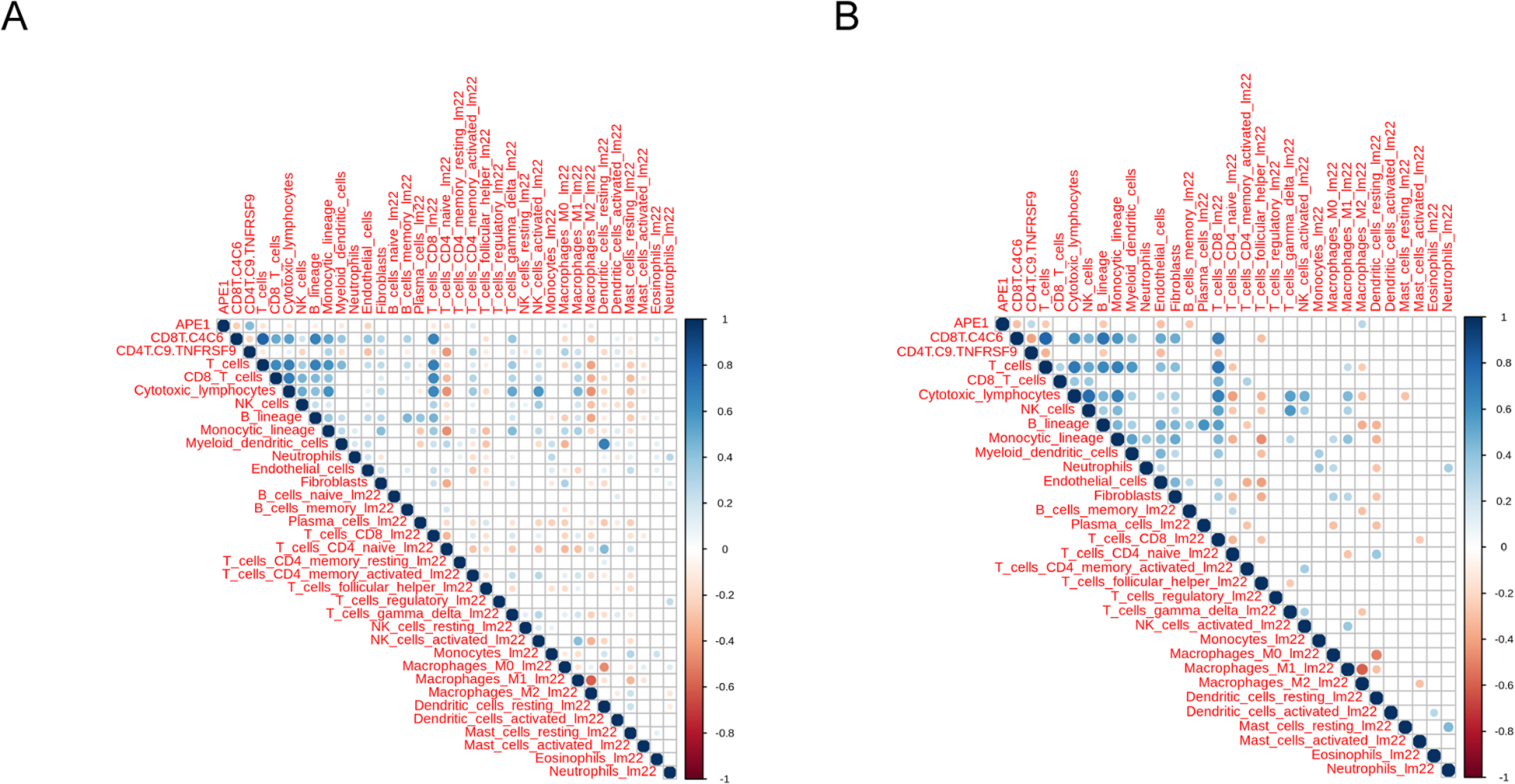
Correlation between APE1 expression and TILs infiltration in GEO database. **a** Correlation between APE1 expression and TILs infiltration in adenocarcinoma subset (*n* = 765). **b** Correlation between APE1 expression and TILs infiltration in squamous cell subset (*n* = 101)

To explore the relationship between APE1 expression and TILs infiltration, the abundance of TILs was estimated by using two methods separately, MCPcounter [18] and FARDEEP [19]. Spearman correlation analysis showed that there was a weak but significant negative correlation between APE1 expression and the abundance of NK cells, T cells, B lineage, cytotoxic lymphocytes by MCPcounter algorithm. With a more refined approach, FARDEEP revealed that memory B cells, CD4^+^ naïve T cells and CD8^+^ T cells exhibited faint but significant negative correlation with APE1 expression, whereas activated memory CD4^+^ T cells, follicular helper T cells were positive correlation with APE1 expression (Table 2). It was worthily noted that the correlation between the expression of APE1 with CD8-C4-GZMK/CD8-C6-LAYN ratio and TNFRSF9^+^ Treg was more pronounced (Table 2). The positive and negative correlation between the expression of APE1 and TNFRSF9^+^ Treg and CD8-C4-GZMK/CD8-C6-LAYN ratio were consistent in both adenocarcinoma subset and squamous cell subset respectively (Fig. 1). However, the negative correlation of the expression of APE1 and CD4^+^ native T cells was only observed in the adenocarcinoma subset (Table 2). In addition, we further evaluated the abundance of TILs of our own 20 NSCLC tissues according to read count. Because CIBERSORT is originally derived from microarray profile data and not suitable for RNA sequencing data, EPIC algorithm was used to perform this procedure. As shown in Table 3, our data confirmed that the abundance of CD4^+^ T cells was clearly significantly negative related to APE1 expression and TNFRSF9^+^ Treg was strongly positively associated with expression of APE1. All together, we assumed that CD4^+^ T cells infiltration may be correlation with APE1 expression, and in particularly TNFRSF9+ Treg which belongs to CD4^+^ T cells influence prognosis in NSCLC given their biological functions during tumor progression.

**Table 2:** The correlation between APE1 and tummor infiltrating lymphocytes in NSCLC in merged GEO databases

MCPcounter			FARDEEP		
TILs	rho	*P* value	TIICs	rho	*P* value
NK cells	−0.164	< 0.001	T cells CD4 memory activated	0.154	< 0.001
T cells	−0.139	< 0.001	B cells memory	−0.134	< 0.001
B lineage	−0.120	< 0.001	T cells follicular helper	0.130	< 0.001
Cytotoxic lymphocytes	−0.089	0.005	T cells CD4 naive	−0.122	< 0.001
CD8 T cells	−0.034	0.282	T cells CD8	−0.108	< 0.001
scRNAseq signatures			NK cells resting	−0.055	0.083
	rho	*P* value	T cells CD4 memory resting	−0.039	0.216
CD8-C4-GZMK/CD8-C6-LAYN ratio	−0.270	< 0.001	T cells gamma delta	−0.023	0.473
TNFRSF9+ Treg	0.386	< 0.001	B cells naive	0.012	0.710
			NK cells activated	−0.012	0.708
			T cells regulatory	−0.006	0.862

*TILs* tumor infiltrating lymphocytes, *rho* Spearman’s rank correlation

**Table 3:** The correlation between APE1 and tummor infiltrating lymphocytes in 20 patients

TILs	Estimate.rho	*P* value
CD4^+^ T cells	−0.526	0.019
NK cells	−0.379	0.100
other Cells	0.349	0.132
B lineage	−0.308	0.186
CD8^+^ T cells	0.260	0.267
B cells	−0.198	0.400
NK cells	0.155	0.513
Cytotoxic lymphocytes	−0.141	0.551
CD8^+^ T cells	−0.137	0.564
T cells	−0.056	0.816
CD8-C4-GZMK/CD8-C6-LAYN ratio	−0.397	0.084
TNFRSF9^+^ Treg	0.671	0.002

*TILs* tumor infiltrating lymphocytes

### Relationship of APE1 expression, CD4^+^ naïve T cells infiltration with NSCLC prognosis

To understand the relationship of APE1 expression, CD4^+^ naïve T cells infiltration with NSCLC prognosis. First, we evaluated the effect of baseline clinical characteristics including sex (Male vs Female), age, histology (Squamous vs Adenocarcinoma and Others vs Adenocarcinoma) and clinical stages (II vs I and III + IV vs I) on relapse-free survival (RFS) of 1006 patients in GEO databases. The results indicated that age (HR = 1.015, 95% CI: 1.001–1.021, *P* < 0.001) and stage (II vs. I HR = 2.496, 95% CI: 1.992–3.128, *P* < 0.001; III vs. I HR = 3.913, 95% CI: 2.976–5.147, *P* < 0.001) were independent prognostic factors for RFS.

Next, univariate Cox regression analysis showed that APE1 was a significant prognostic risk factor for RFS, implying that the higher the expression of APE1, the higher the risk of recurrence (HR = 1.410, 95% CI: 1.130–1.760, *P* = 0.002). Conversely, the abundance of CD4^+^ naïve T cells was an protective factor, implying that the higher abundance of CD4^+^ naïve T cells, the lower the recurrence risks (HR = 0.101, 95% CI: 0.021–0.474, *P* = 0.004). In addition, TNFRSF9^+^ Treg and CD8-C4-GZMK/CD8-C6-LAYN ratio were strongly prognostic factors for RFS. APE1 expression and the abundance of CD4^+^ naïve T cells trended to be significantly associated with RFS in multivariate Cox regression adjusted for age, sex, histology and clinical stage (APE1: *P* = 0.044; CD4^+^ naïve T cells: *P* = 0.086) (Table 4). In the whole population, multivariate Cox regression revealed that only age (HR = 1.027. 95% CI: 1.017–1.038, *P* < 0.001), clinical stage (II vs I HR = 2.406, 95% CI: 1.908–3.035; III + IV vs I HR = 3.473 95% CI: 2.591–4.655, *P* < 0.001), abundance of CD4 T cells (HR = 0.140, 95% CI: 0.023–0.862, *P* = 0.034) and CD8-C4-GZMK/CD8-C6-LAYN ratio (HR = 0.347, 95% CI: 0.161–0.746, *P* = 0.007) were independent prognostic factors for RFS.

**Table 4:** The result of COX analysis of APE1 and the abundance of TILs with RFS in GEO databases

	HR(95% CI)	*P*	HR(95% CI)*	P*
APE1	1.410 (1.130–1.760)	0.002	1.270 (1.007–1.603)	0.044
T cells CD4 naive	0.101 (0.021–0.474)	0.004	0.243 (0.048–1.224)	0.086
T cells CD8	0.217 (0.031–1.530)	0.125		
B cells memory	0.007 (0.000–5.560)	0.146		
T cells	0.871 (0.697–1.090)	0.226		
NK cells	1.280 (0.860–1.900)	0.226		
T cells follicular helper	2.890 (0.425–19.700)	0.278		
B lineage	0.942 (0.842–1.050)	0.296		
Cytotoxic lymphocytes	1.020 (0.866–1.210)	0.779		
NK cells activated	0.899 (0.048–16.900)	0.943		
CD8-C4-GZMK/CD8-C6-LAYN ratio	0.282 (0.138–0.577)	0.001		
TNFRSF9^+^ Treg	2.140 (1.244–3.683)	0.006		

*Adjusted by sex (Male vs Female), age and clinical stage (II vs I and III + IV vs I) and histology (Squamous vs Adenocarcinoma and Others vs Adenocarcinoma)

### APE1 expression influence the relationship between CD4^+^ T cells infiltration and prognosis in NSCLC patients

**Fig. 2:**
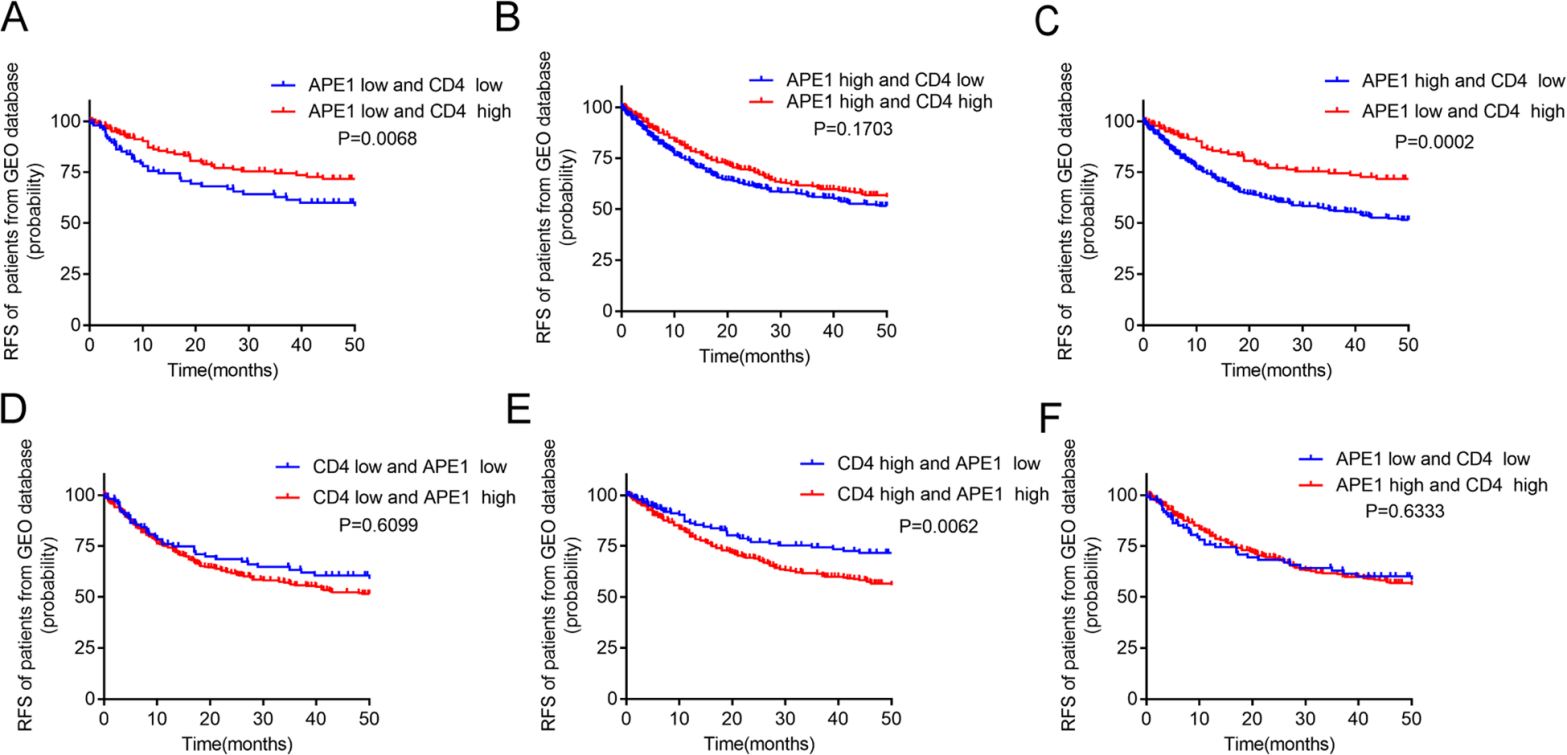
Kaplan-Meier curves for RFS in different groups of NSCLC patients from GEO database. **a** Patients of low APE1 expression and low CD4^+^ naïve T cells infiltration compared with patients of low APE1 expression and high CD4^+^ naïve T cells infiltration. **b** Patients of high APE1 expression and low CD4^+^ naïve T cells infiltration compared with patients of high APE1 expression and high CD4^+^ naïve T cells infiltration. **c** Patients of high APE1 expression and low CD4^+^ naïve T cells infiltration compared with patients of low APE1 expression and high CD4^+^ naïve T cells infiltration. **d** Patients of low CD4^+^ naïve T cells infiltration and low APE1 expression compared with patients of low CD4^+^ naïve T cells infiltration and high APE1 expression. **e** Patients of high CD4^+^ naïve T cells infiltration and low APE1 expression compared with patients of high CD4^+^ naïve T cells infiltration and high APE1 expression. **f** Patients of low APE1 expression and low CD4^+^ naïve T cells infiltration compared with patients of high APE1 expression and high CD4^+^ naïve T cells infiltration

To investigate the influence of different APE1 expression with different abundance of CD4^+^ naive T cells on the prognosis of NSCLC patients. Patients were subsequently divided into different groups according to the 25% percentile of APE1 expression level and the median value of CD4^+^ naïve T cells abundance. The RFS of each group was estimated by Kaplan-Meier and the differences were examined by log-rank. As shown in Fig. 1, the patients with high CD4^+^ naive T cells abundance had significantly longer RFS only in the low APE1 expression groups(*P* < 0.01; Fig. 2a, c and e). However, CD4^+^ T cells abundance was no more a favorable factor for longer RFS in the high APE1 expression groups (*P* > 0.05; Fig. 2b, d and f). These results suggest that high APE1 expression may cripple the beneficial effects of CD4^+^ T cells abundance on RFS.

**Fig. 3:**
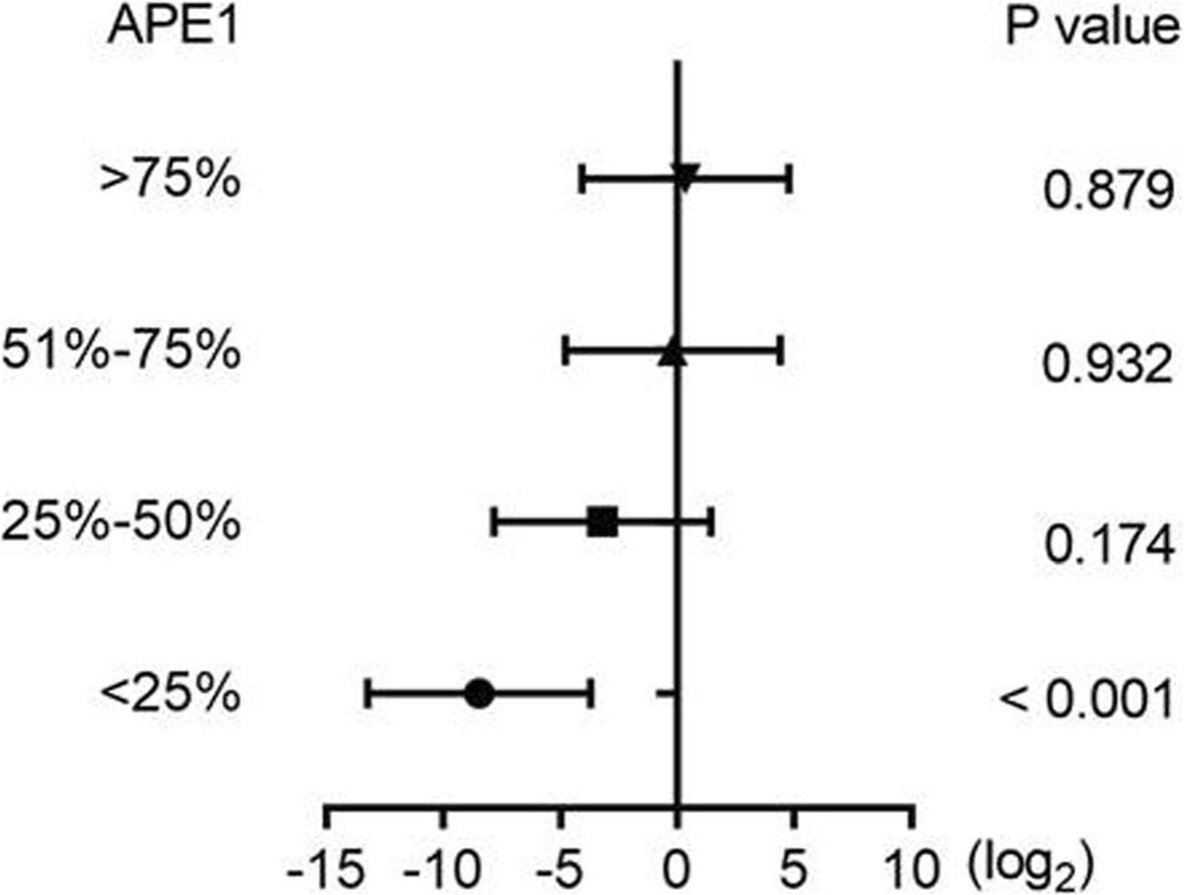
Forest plot illustrating the effects of APE1 percentile grouping on the prognosis of abundance of CD4^+^ T cells for RFS. Patients were divided into different groups according to every increment of 25% percentile of APE1 expression level. When APE1 level was less than 25%, the abundance of CD4^+^ T cells was a favorable prognostic factor (*P* < 0.001), however, with the increase of APE1 level, the impact of CD4 + T cells on prognosis was gradually weakened (*P* > 0.05)

Cox analysis further revealed that the abundance of CD4^+^ naïve T cells was obviously related to RFS in APE1 low expression group in both univariate and multivariate analysis (HR = 0.003, 95% CI: 0.000–0.076, *P* < 0.001). However, there was no significant correlation between the abundance of CD4^+^ naïve T cells and RFS in APE1 high expression group (HR = 0.455, 95% CI: 0.074–2.792, *P* = 0.395). In addition, the interaction between abundance of CD4^+^ naïve T cells and APE1 expression level reached statistical significance (*P* = 0.013), and persisted after adjusted for sex, age and clinical stage. The forest plot further confirmed that the favorable effect of the abundance of CD4^+^ naïve T cells on RFS was deteriorated with elevated APE1 expression level (Fig. 3, Table 5). In contrast, although both TNFRSF9^+^ Treg and CD8-C4-GZMK/CD8-C6-LAYN ratio were prognostic factors in APE1 high expression subgroup not in the APE1 low expression subgroup, no significant interaction was found (Table 5). All together, these results strongly suggest that APE1 may affect the relationship between abundance of CD4^+^ naïve T cells and prognosis, and the patients with low APE1 expression plus high CD4^+^ naïve T cells infiltration displayed most significant survival benefits.

**Table 5:** The result of Cox analysis evaluating prognosis of CD4+ naive T cells for RFS in different APE1 level subgroups in GEO databases

	CD4^+^ naive T cells			
	HR (95% CI)	*P*	HR (95% CI)*	P*
Low APE1	0.003 (0.000–0.076)	< 0.001	0.009 (0.000–0.361)	0.013
High APE1	0.455 (0.074–2.792)	0.395	0.604 (0.098–3.847)	0.604
Interaction	117.46 (2.725–5062)	0.013	59.299 (1.246–2822)	0.038
	CD8^+^T C4/C6 ratio	*P*	TNFRSF9^+^ Treg	*P*
	HR (95% CI)		HR (95% CI)	
Low APE1	0.415 (0.078–2.221)	0.304	0.825 (0.246–2.764)	0.756
High APE1	0.298 (0.136–0.656)	0.003	2.212 (1.103–4.080)	0.024
Interaction	0.846 (0.134–5.349)	0.859	2.257 (0.576–8.850)	0.243

*Adjusted by gender (Male vs Female), age, histology (Squamous vs Adenocarcinoma and Others vs Adenocarcinoma) and clinical stage (II vs I and III + IV vs I)

### APE1 expression and CD4^+^ T cells infiltration in tissue samples and the associations with prognosis

**Fig. 4:**
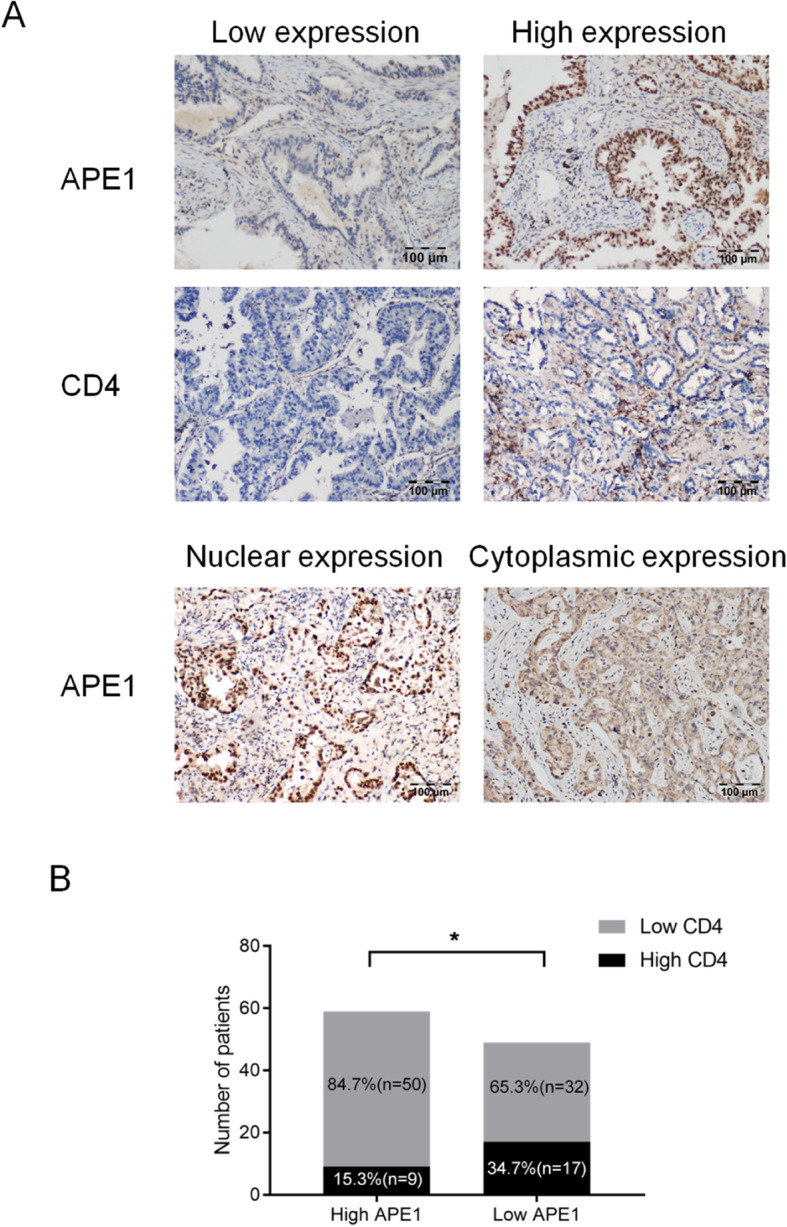
APE1 expression level was associated with the CD4 ^+^ T cell infiltration. **a** The representative IHC images of APE1 and TILs density of CD4^+^ cells. **b** CD4 ^+^ T cell infiltration was negative associated with the APE1 expression level (*P* < 0.05)

To further investigate the association between APE1 expression and the density of tumor-infiltrating CD4^+^ T cell subsets and confirm the results stemmed from the GEO datasets, APE1 expression and CD4^+^ T cell infiltration were divided into high- and low-score groups separately according to the IHC staining in the other cohort comprised of 108 NSCLC patients. The basic information of the included patients is shown in Table 6. The representative IHC images of APE1 and TILs density of CD4^+^ cells are shown in Fig. 4a. As shown in Fig. 4b, 50/59 APE1-high patients showed low CD4^+^ T cells infiltration, while 32/49 APE1-low patients showed low CD4^+^ T cells infiltration (*P* < 0.05, Chi-square test), this suggests that CD4^+^ T cell infiltration was negatively associated with the APE1 expression level.

**Table 6:** Patients’ basic characteristic of 108 NSCLC patients

Characteristic	The number of patients, n (%)
Age	57.97 ± 8.36
Age range	
≥ 60 years	52 (48.15)
< 60 years	56 (51.85)
Sex	
Male	54 (50.00)
Female	54 (50.00)
Smoking status	
ever	49 (45.37)
never	59 (54.63)
Histological cell type	
Adenocarcinoma	108 (100.00)
Tumor stage	
I	107 (99.07)
II	1 (0.93)

**Fig. 5:**
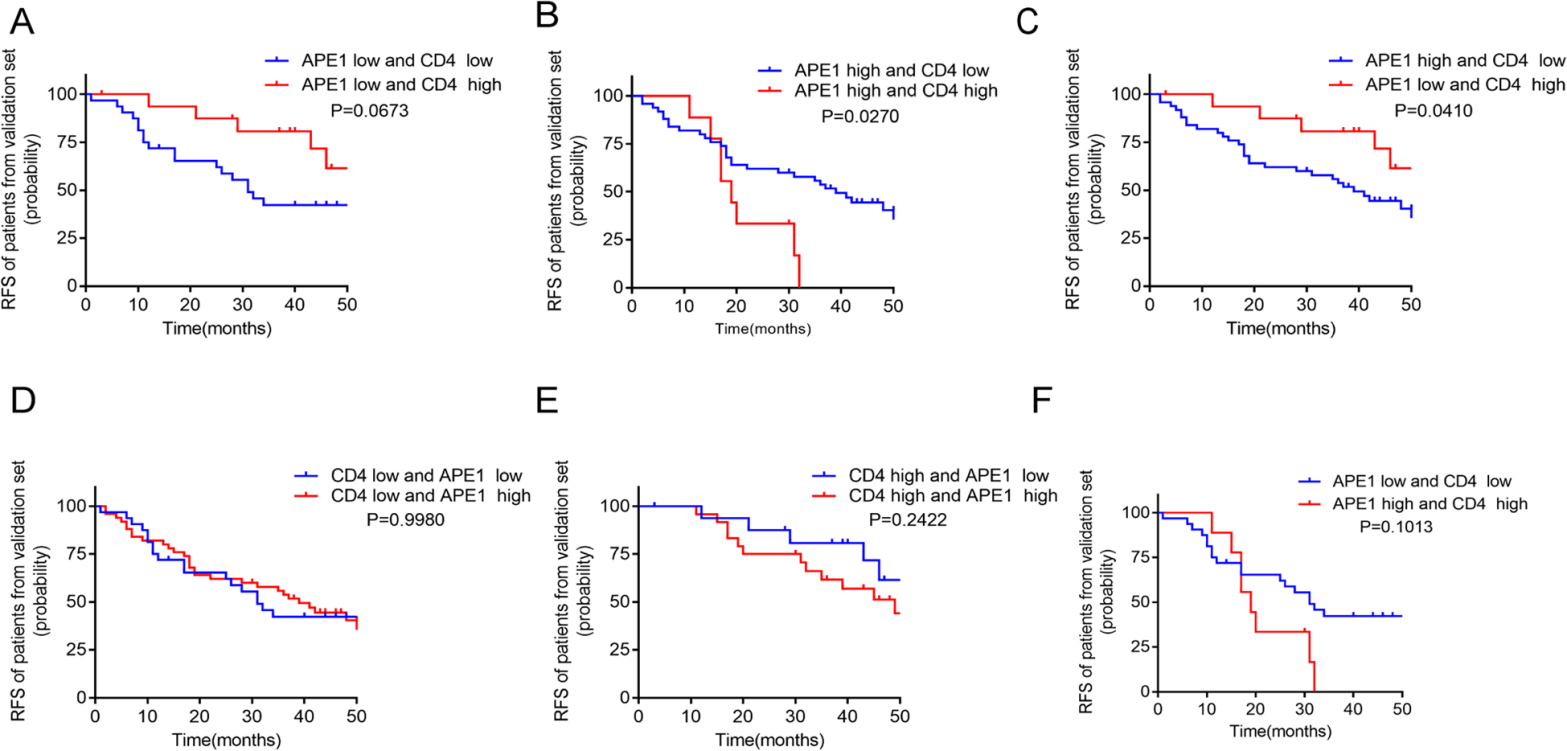
Kaplan-Meier curves for RFS in differernt groups of patients from validation cohort. **a** Patients of low APE1 expression and low CD4^+^ T cells infiltration compared with patients of low APE1 expression and high CD4^+^ T cells infiltration. **b** Patients of high APE1 expression and low CD4^+^ T cells infiltration compared with patients of high APE1 expression and high CD4^+^ T cells infiltration. **c** Patients of high APE1 expression and low CD4^+^ T cells infiltration compared with patients of low APE1 expression and high CD4^+^ T cells infiltration. **d** Patients of low CD4^+^ T cells infiltration and low APE1 expression compared with patients of low CD4^+^ T cells infiltration and high APE1 expression. **e** Patients of high CD4^+^ T cells infiltration and low APE1 expression compared with patients of high CD4^+^ T cells infiltration and high APE1 expression. **f** Patients of low APE1 expression and low CD4^+^ T cells infiltration compared with patients of high APE1 expression and high CD4^+^ T cells infiltration

Next, we investigated the effect of APE1 expression and CD4^+^ T cells infiltration on prognosis in NSCLC patients. Consistent with findings from GEO database, we found the same trend with all groups (Fig. 5). In particular, low APE1 expression and high CD4^+^ T cells infiltration was associated with longer RFS in the NSCLC patients (Fig. 5a and c). Intriguingly, we found that high CD4^+^ T cells infiltration was even significantly related with the poor prognosis in the high APE1 expression group (*P* = 0.027, Fig. 5b). Furthermore, the interaction test showed that the interaction between abundance of CD4^+^ T cells infiltration and APE1 expression level was significant (*P* = 0.016). Based on these result, we further confirmed that APE1 expression level may influence the associations of CD4^+^ T cells infiltration with the prognosis in NSCLC patients.

## Discussion

NSCLC, as a tumor with a high incidence and mortality, has become a great threat to human health due to its limited and complex treatment [24]. Nowadays, it has been repeatedly clarified that tumor immune microenvironment carrys a considerable weight in the evaluation of prognosis and immunotherapy of NSCLC patients [25]. Immunotherapy drugs represented by immune check-point inhibitors, as an emerging therapeutic method, have attracted more and more attention in the field of cancer research.

As a multifunctional protein of the BER-pathway, APE1 has been found to be involved in DNA repair of oxidative base damage, redox regulation of transcription factors, and closely related to RNA metabolism. Association of dysregulation of APE1 with various human pathologies, such as cancer, cardiovascular diseases and neurodegeneration, is attributable to its multifunctional nature, and this makes APE1 a potential therapeutic target [26]. Our study found for the first time that APE1 was negative related to CD4^+^ T cells infiltration, which provides new perspectives for exploring new roles of this versatile protein with a wide variety of functions. Compelling evidence proved that DNA damage repair reaction and immune reaction jointly maintain the internal enviroment balance of organism [27]. As a key enzyme of intracellular base excision and repair pathway, it is highly probable that APE1 may be involved in such interaction within the tumor microenvironment. Studies of mesenchymal cells or tumor cells have demonstrated that surface adhesion receptors, such as CD44, plays an important role of in the migratory cycle of T cells [28], while APE1 can cleave CD44 mRNA in vitro studies through exhibiting its endonucleases function [29]. Moreover, the effect of APE1 on immune cells, such as T cells, B cells and monocyte macrophages, has also been widely studied [14, 15, 30]. Given the aforementioned findings, it is not surprising that APE1 was associated with the abundance of CD4^+^ T cells.

Most studies believed that high tumor CD4^+^ T cells infiltration usually indicates a favourable prognosis. Consistently, our study found that the high abundance of CD4^+^ T cells was associated with better prognosis. Interestingly, according to our analysis, it appears that it is possible to counteract this favourable prognostic effect by increasing APE1 expression. Compared with patients with low APE1 expression, the abundance of CD4^+^ T cells was no longer an indicator of good prognosis in patients with high APE1 expression. We speculate that the underlying mechanism involved might be due to the regulatory effects of APE1 on the function of CD4^+^ T cells. A study confirmed that APE1 can inhibit the release of IL-12 from antigen-presenting cells and thus diminish the T helper cell type 1 responses induced by IL-12 [15], and the authors strongly suggest that redox function of APE1 plays a major role in this process. Research has showed that transcription factors including members of the nuclear factor of activated T cell (NFAT), NF-κB, and activator protein-1 (AP-1) families are important during the process of T cells differentiation [31]. Therefore, it is reasonable to believe that APE1 may be involved in T cells differentiation by regulating these transcription factors. Furthermore, a considerable number of studies together showed that APE1 directly regulated the expression of IL-6 and IL-8 through its redox function [32–34], which also may contribute to the inhibitory immune microenvironment and lead to unfavorable prognosis in NSCLC patients.

It is believed that interaction of various factors is associated with the prognosis of patients with NSCLC. Some studies have shown that the high CD4^+^ T cell infiltration is a favorable prognosis factor of NSCLC [35], whereas other studies suggested that high CD4^+^ T cell infiltration was not associated with the better prognosis [36]. What we found in this research implies that when APE1 high expression in NSCLC patients, even with CD4^+^ T cell high infiltration, it may also indicate an unfavorable prognosis. The present study provides a novel perspective to explain this phenomenon. However, we did not analyze the impact of CD4 ^+^ T and APE1 on prognosis in specific treatment, such as adjuvant TKI therapy. Therefore, it is difficult to judge whether CD4 ^+^ T and APE1 can be used as predictive biomarkers for specific treatment from the current study. Due to the small sample size and a limited number of tissue samples available, lung tissues were not subjected to immunohistochemical analysis of distinct T cell subsets, which result in some slight differences between GEO database and our IHC results. To this end, further studies are necessarily required to elucidate the detailed molecular mechanisms by which APE1 regulates CD4^+^ T cells in NSCLC.

## Conclusion

In conclusion, we have identified APE1 may affect the relationship between CD4^+^ T cells infiltration and prognosis in NSCLC. Our study suggested that APE1 combined with CD4^+^ T cells might be a potential predictor of outcome in patients with NSCLC, and combining immunotherapy and APE1-targeted therapy may be a promising treatment for NSCLC.

## Supplementary Information

**Additional file 1 **: **Table S1.** Subcellular localization of APE1 of 118 NSCLC samples.

**Additional file 2. **APE1 expression and immune cell population in 20 NSCLCs.

**Additional file 3. **APE1 expression and immune cell population in GEO database.

## Data Availability

For GEO database, the data can be obtained from the Gene Expression Omnibus (GEO) public database under accession number GSE68465, GSE30219, GSE31210 and GSE50081. The data of APE1 expression and immune cell population in 20 NSCLCs can be obtained from supplementary file.

## Abbreviations

APE1: Apurinic/apyrimidinic endonuclease 1
TILs: Tumor infiltrating lymphocytes
NSCLC: Non-small cell lung cancer
GEO: Gene Expression Omnibus
RFS: Recurrence free survival
IHC: Immunohistochemistry
