# Supplementary material for: APE1 may influence CD4+ naïve T cells on recurrence free survival in early stage NSCLC
Source: BMC Cancer. 2021 Mar 6;21:233. doi: 10.1186/s12885-021-07950-1 (PMC7937314; doi:10.1186/s12885-021-07950-1)
Supplement: Supplementary file 1 — Additional file 1 : Table S1. Subcellular localization of APE1 of 118 NSCLC samples. [file 12885_2021_7950_MOESM1_ESM.docx]

| **S. Table 1** Subcellular localization of APE1 of 118 NSCLC samples | | | | | | | |
| --- | --- | --- | --- | --- | --- | --- | --- |
| Samples | APE1  score | CD4  score | APE1 Location | Samples | APE1  score | CD4  score | APE1 Location |
| 1 | Low | High | None | 55 | High | Low | nuclear |
| 2 | Low | Low | Cytoplasm | 56 | High | Low | Cytoplasm |
| 3 | Low | Low | Cytoplasm | 57 | High | Low | nuclear |
| 4 | Low | High | Cytoplasm | 58 | High | Low | nuclear |
| 5 | Low | Low | Cytoplasm | 59 | High | High | Cytoplasm |
| 6 | Low | Low | Cytoplasm | 60 | High | Low | Cytoplasm |
| 7 | Low | Low | Cytoplasm | 61 | High | Low | Cytoplasm |
| 8 | Low | Low | None | 62 | High | High | Cytoplasm |
| 9 | Low | High | Cytoplasm | 63 | High | Low | Cytoplasm |
| 10 | Low | Low | Cytoplasm | 64 | High | High | Cytoplasm |
| 11 | Low | High | Cytoplasm | 65 | High | Low | Cytoplasm |
| 12 | Low | Low | Cytoplasm | 66 | High | High | Cytoplasm |
| 13 | Low | Low | None | 67 | High | Low | nuclear |
| 14 | Low | High | Cytoplasm | 68 | High | Low | nuclear |
| 15 | Low | High | None | 69 | High | Low | nuclear |
| 16 | Low | High | Cytoplasm | 70 | High | High | Cytoplasm |
| 17 | Low | High | None | 71 | High | Low | nuclear |
| 18 | Low | Low | Cytoplasm | 72 | High | Low | Cytoplasm |
| 19 | Low | Low | None | 73 | High | High | Cytoplasm |
| 20 | Low | Low | Cytoplasm | 74 | High | Low | Cytoplasm |
| 21 | Low | High | Cytoplasm | 75 | High | Low | Cytoplasm |
| 22 | Low | Low | Cytoplasm | 76 | High | High | Cytoplasm |
| 23 | Low | Low | nuclear | 77 | High | Low | Cytoplasm |
| 24 | Low | Low | Cytoplasm | 78 | High | High | Cytoplasm |
| 25 | Low | Low | None | 79 | High | Low | Cytoplasm |
| 26 | Low | Low | None | 80 | High | High | Cytoplasm |
| 27 | Low | High | None | 81 | High | Low | Cytoplasm |
| 28 | Low | Low | Cytoplasm | 82 | High | Low | Cytoplasm |
| 29 | Low | Low | None | 83 | High | Low | Cytoplasm |
| 30 | Low | Low | Cytoplasm | 84 | High | Low | Cytoplasm |
| 31 | Low | High | nuclear | 85 | High | Low | nuclear |
| 32 | Low | Low | nuclear | 86 | High | Low | Cytoplasm |
| 33 | Low | Low | Cytoplasm | 87 | High | Low | Cytoplasm |
| 34 | Low | Low | None | 88 | High | Low | Cytoplasm |
| 35 | Low | High | None | 89 | High | Low | Cytoplasm |
| 36 | Low | High | nuclear | 90 | High | Low | nuclear |
| 37 | Low | Low | nuclear | 91 | High | Low | Cytoplasm |
| 38 | Low | Low | None | 92 | High | Low | Cytoplasm |
| 39 | Low | Low | Cytoplasm | 93 | High | Low | nuclear |
| 40 | Low | High | Cytoplasm | 94 | High | Low | nuclear |
| 41 | Low | Low | Cytoplasm | 95 | High | Low | Cytoplasm |
| 42 | Low | Low | Cytoplasm | 96 | High | Low | Cytoplasm |
| 43 | Low | High | Cytoplasm | 97 | High | Low | nuclear |
| 44 | Low | Low | Cytoplasm | 98 | High | Low | Cytoplasm |
| 45 | Low | High | Cytoplasm | 99 | High | Low | Cytoplasm |
| 46 | Low | High | Cytoplasm | 100 | High | Low | nuclear |
| 47 | Low | Low | None | 101 | High | Low | Cytoplasm |
| 48 | Low | Low | Cytoplasm | 102 | High | Low | Cytoplasm |
| 49 | Low | Low | nuclear | 103 | High | Low | Cytoplasm |
| 50 | High | Low | Cytoplasm | 104 | High | Low | nuclear |
| 51 | High | Low | Cytoplasm | 105 | High | Low | Cytoplasm |
| 52 | High | Low | Cytoplasm | 106 | High | Low | Cytoplasm |
| 53 | High | Low | Cytoplasm | 107 | High | Low | Cytoplasm |
| 54 | High | Low | nuclear | 108 | High | Low | Cytoplasm |
